# Mismeasured mortality: correcting estimates of wolf poaching in the United States

**DOI:** 10.1093/jmammal/gyx052

**Published:** 2017-05-19

**Authors:** Adrian Treves, Kyle A Artelle, Chris T Darimont, David R Parsons

**Affiliations:** 1 Nelson Institute for Environmental Studies, University of Wisconsin–Madison, 30A Science Hall, 550 North Park Street, Madison, WI 53706, USA (AT); 2 Earth2Ocean Research Group, Department of Biological Sciences, Simon Fraser University , 8888 University Drive, Burnaby, British Columbia V5A 1S6, and Hakai Institute and Raincoast Conservation Foundation, Canada (KAA); 3 Department of Geography, University of Victoria, P.O. Box 1700, Stn CSC, Victoria, British Columbia V8W 2Y2, and Hakai Institute and Raincoast Conservation Foundation, Canada (CTD); 4 Project Coyote, 2723 Decker Avenue NW, Albuquerque, NM 87107, USA (DRP)

**Keywords:** carnivore, endangered species, illegal, lethal control, mark–recapture, regulated take

## Abstract

Measuring rates and causes of mortalities is important in animal ecology and management. Observing the fates of known individuals is a common method of estimating life history variables, including mortality patterns. It has long been assumed that data lost when known animals disappear were unbiased. We test and reject this assumption under conditions common to most, if not all, studies using marked animals. We illustrate the bias for 4 endangered wolf populations in the United States by reanalyzing data and assumptions about the known and unknown fates of marked wolves to calculate the degree to which risks of different causes of death were mismeasured. We find that, when using traditional methods, the relative risk of mortality from legal killing measured as a proportion of all known fates was overestimated by 0.05–0.16 and the relative risk of poaching was underestimated by 0.17–0.44. We show that published government estimates are affected by these biases and, importantly, are underestimating the risk of poaching. The underestimates have obscured the magnitude of poaching as the major threat to endangered wolf populations. We offer methods to correct estimates of mortality risk for marked animals of any taxon and describe the conditions under which traditional methods produce more or less bias. We also show how correcting past and future estimates of mortality parameters can address uncertainty about wildlife populations and increase the predictability and sustainability of wildlife management interventions.

An accurate understanding of causes of death in animal populations is important for effective management and legitimate policy. Contemporary study of wild animal populations has benefited enormously from mark–recapture methods to estimate life history variables, such as mortality. However, marked animals in such studies sometimes elude recapture, which leads to loss of data (i.e., unknown fates). When the proportion of unknown fates among marked animals is low, the potentially biasing effects of data loss might be correspondingly low. Also, when the mortality risks for unknown fates are very similar to those for known fates, the loss of data should not bias the estimates of life history traits (i.e., this would be “uninformative censoring”). The traditional presumptions in most studies are that marked animals disappear because they moved out of range of telemetry or the transmitter technology affixed to the animal failed, but otherwise researchers assume the life and death of those animals proceeded as it would otherwise have done. We examine this assumption for wolves (*Canis lupus* and *C. rufus*) in the United States, and emerge with a generalizable insight broadly applicable to many taxa.

Although early research on grizzly bears (*Ursus arctos*) suggested data loss was biased when humans destroyed radiotransmitters (McLellan et al. 1999), this idea was not quantified for gray wolves (*C. lupus*) until study of the mortality and poaching of Scandinavian wolves (Liberg et al. 2012). When Adams et al. (2008) documented that 74% of human-caused deaths went unreported in an Alaskan gray wolf population, even that high rate of loss of data on wolves did not raise concerns, perhaps because unreported killing seemed inconsequential to a large, resilient wolf population. Later, parallel analyses of Northern Rocky Mountain (NRM) gray wolves appeared to accept the assumption of uninformative censoring (Murray et al. 2010). They cited unpublished analyses showing that including dead radiocollared wolves for which cause of death could not be inferred did not produce “qualitatively different results” (Murray et al. 2010:2517). Those unpublished analyses of recovered marked wolves whose cause of death was unknown are not peer-reviewed as of the time of writing. That same year, some of the same authors published another mortality analysis (Smith et al. 2010), in which they inferred that some marked wolves of unknown fates dispersed and eluded telemetry, because the proportion of suspected dispersers that disappeared (31.4%) differed from the proportion (18.1%) of known residents that disappeared. High-altitude aerial telemetry conducted intensively across the recovery areas was oriented to locating dispersers because of the importance of such events (Bangs and Fritts 1996). Smith et al. (2010) analyzed the last known locations prior to disappearances to infer that modestly informative censoring was present and the locations of disappearance were not in areas of high human activity, therefore “associated principally with dispersal status rather than human-caused mortality” (Smith et al. 2010:632). That inference hinges on the hypothesis that levels of poaching would be higher in areas of higher human activity. However, we suggest that strict protection of wolves might alternatively have made people reluctant to kill a wolf where the likelihood of witnesses seemed higher. If so, locations more prone to poaching might instead include more remote areas. Remote hunting zones might reasonably be implicated given that recent research on inclination to poach indeed implicates hunters in both the NRM and in the state of Wisconsin (Treves and Martin 2011; Treves et al. 2013; Treves et al. 2017a). After Liberg et al. (2012), attention to poaching grew in the wolf research community.

Studying Scandinavian gray wolves, researchers estimated the major cause of death was poaching, which accounted for 51% of all mortality (poaching risk). An estimated 66% of that poaching went unreported (Liberg et al. 2012). Because the study reconstructed the fates of poached wolves that went missing, it drew attention to—and undermined—the previously held assumptions that a small proportion of marked animals disappeared and that data loss was minimal. It also raised questions about the assumption that unknown fates resembled known fates in mortality risk and rate (i.e., censoring was informative in the Scandinavian study). Further evidence of a problem with the latter assumption followed reanalysis of data from Adams et al. (2008), working in the Brooks Range of Central Alaska. Schmidt et al. (2015) reported at least 15% higher mortality among unmarked gray wolves compared to their marked pack-mates. In contrast, another Alaskan study around Denali National Park and Preserve reported that marked wolves suffered higher rates of regulated killing (Borg et al. 2016). These study sites in Alaska, however, differed. The former had few roads, and few people, whereas the latter had more of both suggesting that the relative risk from humans for marked and unmarked animals might be influenced by whether humans can detect collars and are killing wolves legally. A study in Wisconsin, across a landscape with denser human activity including many roads, people, livestock, hunters, hounds, etc., produced an estimated 28% higher mortality rate for unmarked gray wolves than for marked wolves when illegal killing comprised almost half of all deaths (Treves et al. 2017b). Despite current uncertainty about why marked or unmarked wolves face different rates of mortality from humans in different systems, all these studies converge to suggest that the traditional assumption is unsupported: fates of marked wolves do not seem to accurately represent the risk and rates of mortality for the broader population.

Based on the above, we test whether unknown fates of marked wolves cause important losses of information that would bias results. We also test the specific hypothesis that poaching is systematically underestimated when data from wolves of unknown fates are omitted. We reanalyzed data from 4 populations of wolves in the United States (2 populations of gray wolves, *C. lupus*; 1 population of Mexican gray wolves, *C. l. baileyi*; and 1 population of red wolves, *C. rufus*). Although our results are specific to wolves, we identify a general mechanism that applies to studies of other species whose mortality can be divided into deaths where the cause is known and deaths where the cause is unknown.

## Materials and Methods

We define legal killing to include regulated harvest or government removal of a protected animal, as long as the death was reported after a permitted activity. We define poaching as any non-permitted killing in which the actor intended to kill an animal (trapping, poison, shooting, etc.), as opposed to most vehicle collisions in which the driver likely does not intend to kill any animal. This definition of poaching is justified under the Endangered Species Act because the U.S. Congress of 1973 explicitly made it illegal to kill a listed species regardless of “knowingly” doing so (Newcomer et al. 2011). Also, we redefine “known fates” and “unknown fates” from their common usage for marked animals. We define known fate as any marked animal whose cause of death is confirmed (i.e., excluding marked animals whose remains are recovered but are assigned to “unknown cause” of death, and excluding marked animals that disappear). Importantly, we differ from several other authorities by highlighting that “unknown cause” of death never includes legal killing (because, by definition, a legal kill must be reported so its cause is known). Finally, many studies of marked animals have to contend with the possibility that a marked animal that disappeared is still alive but has eluded monitoring. We avoid this difficulty for all 4 populations under analysis by restricting ourselves to older time periods, so radiocollared wolves could not still be alive today.

### 

#### Section 1: calculating the bias in mortality estimates.

**Fig. 1.**
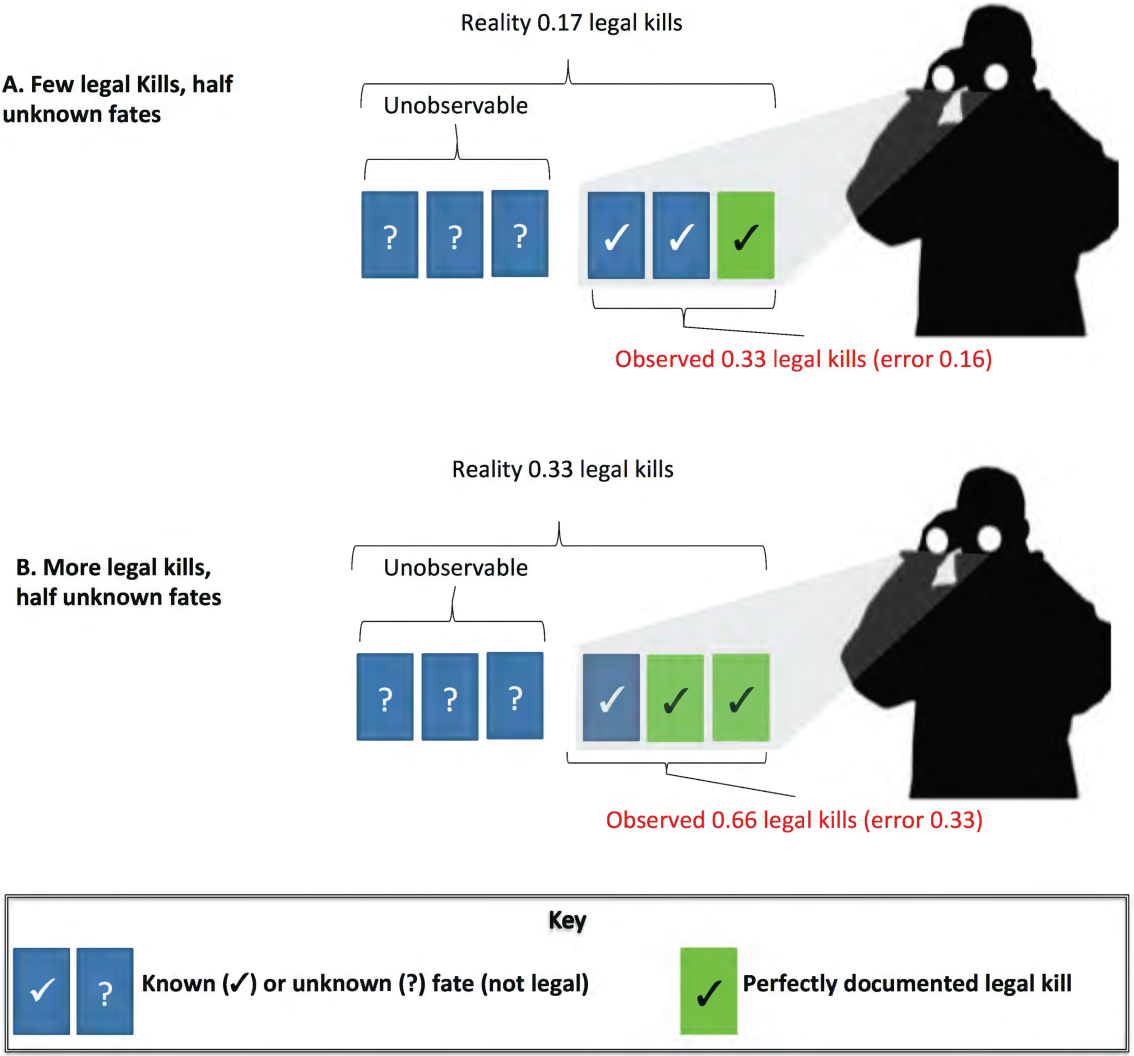
Systematic bias in calculating the risk of mortality from legal killing when some marked animals have unknown fates (unobservable with question marks ?) and causes of death vary in the accuracy of documentation. The green squares represent legal kills (perfectly documented) and the blue squares denote other causes of death (inaccurately documented). Observed (silhouette with binoculars) known fates (check marks ✓, and calculation in red text) alone would overestimate the real risk of legal killing. A) Positive bias in estimating risk of legal killing is 0.16. B) Positive bias increases by 0.17 as the proportion of legal kills increases.

We begin with the mathematics underlying estimation of risk of mortality, defined as the proportion of all deaths attributable to a given cause. The traditional assumption was that data lost from unknown fates was uninformative, because the marked animals with known fates ostensibly represented all marked animals’ fates. This assumed the relative risks of different causes of death were approximately equivalent in marked animals of known and unknown fates. However, marked animals of unknown fate never die from perfectly documented causes, such as legal killing, or they would not have disappeared. Therefore, the animals of known fate cannot represent the animals of unknown fate accurately (Fig. 1A).

The mismatch between animals of known fate and those of unknown fate introduces error that is not random but systematic (biasing). The error is always in the direction of underestimating the risk posed by inaccurately documented causes of death because these sometimes lead to unknown fates. Conversely, overestimation of risks of the perfectly documented causes of death (e.g., legal killing in our context) occurs because these causes are not represented among the unknown fates at all. Therefore, the traditional assumption that marked animals of known fate represent fates of all marked animals is inaccurate as a mathematical fact. The only question that remains is how large the inaccuracy might be. We use the method in Table 1A to estimate how much the risk of legal killing has been overestimated in its proportional contribution to total mortality in endangered wolves.

**Table 1.** Estimating the relative risk of mortality as a proportion of marked animals, when marked animals disappear (unknown fates). A) Equal numbers of known and unknown fates, 1 perfectly documented cause of death (legal killing) and 1 inaccurately documented cause of death. B) The general expression for any *n* known fates and *m* unknown fates with 3 causes of death. Prior values are precise and accurate for *n* (number of known fates), *m* (number of unknown fates), *Legal* (number of marked animals killed legally), *Observed*_non_ (number of marked animals of known fate that died from nonhuman causes), *Observed*_oh_ (number of marked animals of known fate that died from human causes other than legal killing), and *Expected*_non_ + *Expected*_oh_ (the number of marked animals of unknown fate expected dead from nonhuman and other human causes, respectively) sum to *m* but have uncertain values. Unknown fates include recovered carcasses with unknown cause of death. *P* is the number of marked animals of unknown fate expected dead from cryptic poaching following equation 2.

Causes of death	Mortality risk for marked animals		
A)	Known fates (50)	Unknown fates (50)	Known + unknown fates (100)
Perfectly documented legal killing	0.20	0^a^	0.10
Inaccurately documented causes	0.80	1.00	0.90
B)	Known fates (*n*)	Unknown fates (*m*)	Known + unknown fates (*n* + *m*)
Legal killing	*Legal*/*n*	0^a^	*Legal*/(*n* + *m*)
Nonhuman causes	*Observed*_non_/*n*	*Expected*_non_/*m*	(*Observed*_non_ + *Expected*_non_)/(*n* + *m*)
Other human causes	*Observed*_oh_/*n*	(*Expected*_oh_ + *P*)/*m*	(*Observed*_oh_ + *Expected*_oh_ + *P*)/(*n* + *m*)

^a^Legal kills must be reported (all known fates) or they are not legal.

**Fig. 2.**
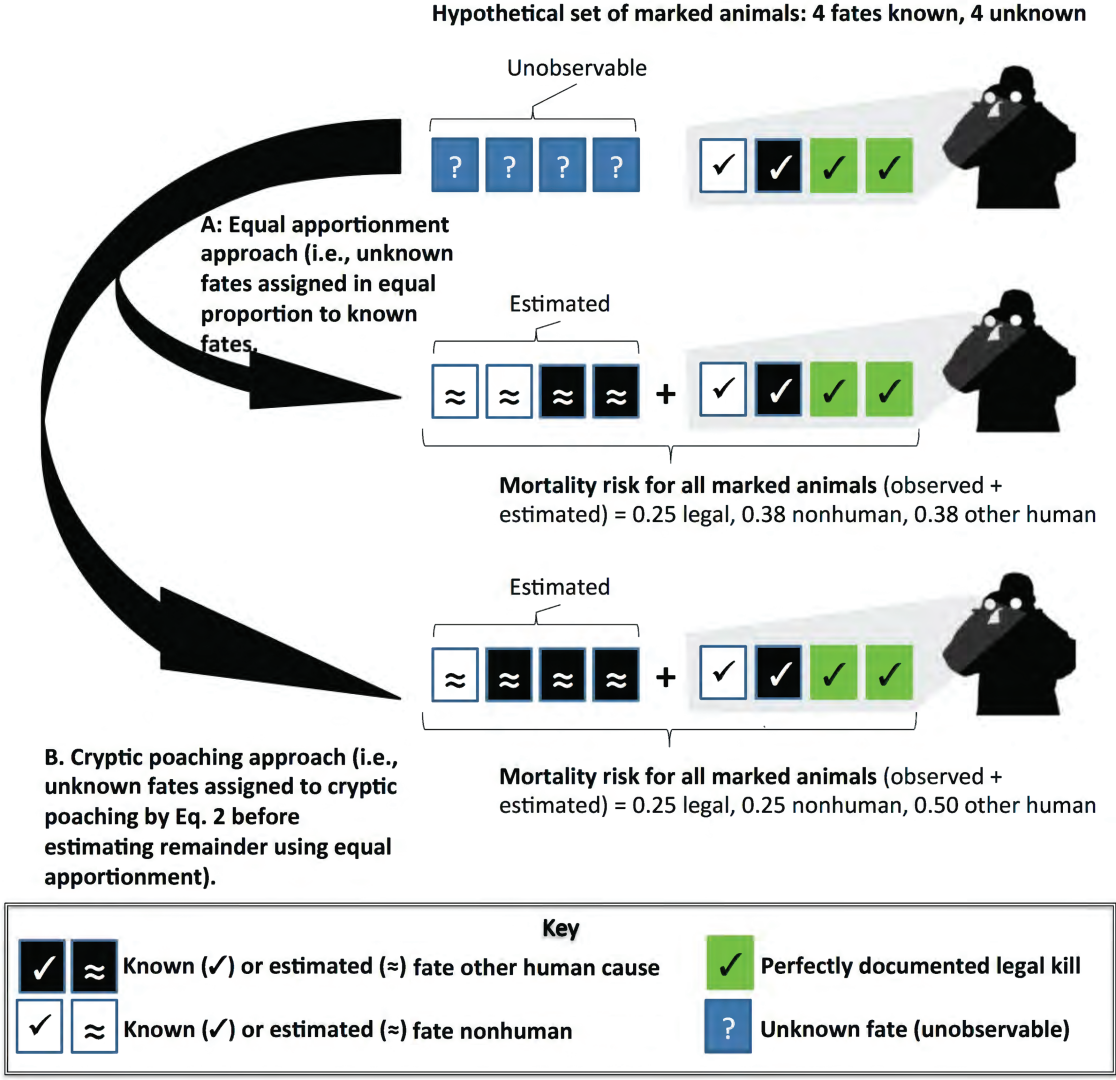
Systematic bias in estimating the risk of mortality when some marked animals have unknown fates (unobservable, question marks ?) and causes of death vary in the accuracy of documentation. Observed (silhouette with binoculars) known fates (check marks ✓) alone would underestimate the inaccurately documented causes of death (unknown fates, white, black, and blue squares). Two approaches to estimating unknown fates produce lower and upper bounds on estimates of risk of mortality, using equations 1a, 1b, and 2. A) The equal apportionment approach assumes that the observed ratio of known nonhuman causes of death (white squares with check marks) to known, other human causes of death (black squares with check marks) applies to the unknown fates (squares with approximately equal signs, ≈). B) The cryptic poaching approach with *C* = 2 from equation 2 assumes that for every 1 known-fate poached animal (black square with check mark) there will be 2 unknown-fate poached animals (black square with ≈), which must be accounted first before equal apportionment of the remainder adds 1 poached and 1 nonhuman cause of death (white square with ≈). This approach requires discrimination between poaching and vehicle collision or other unintentional human causes (see Supplementary Data SD2).

As legal killing increases, the bias caused by discarding information on unknown fates increases (Fig. 1B). As the number of unknown fates (*m*) increases, so too does the bias. The bias increases proportionally to both legal kills and *m* because each additional individual of unknown fate results in increased underestimation of inaccurately documented causes, whereas each additional legal kill results in increased overestimation of the contribution of legal kills. By accounting fully for all marked animals (*n* + *m*) and by estimating the unknown variables (Figs. 2A and 2B; Table 1B), we extract more information from the sample of marked animals than done traditionally. The arithmetic described in Table 1A and Fig. 1 is a mathematical fact. But we can extract yet more information from well-documented cases if we split the causes of death as in Table 1B and consider the role of *P*, which estimates cryptic poaching. Our approach is more efficient because additional information is acquired from the sample of marked individuals.

#### Section 2: estimating unknown fates.

A failure to document death of a marked animal can occur because poachers concealed evidence or because the marked animal eluded monitoring prior to death (Supplementary Data SD1). Eluding monitoring prior to death means a marked animal lived for a time and then died undocumented—at least undocumented by the same method used on marked animals of known fate. It might be reasonable to assume such marked animals are represented well by the known fates, because eluding monitoring does not necessarily imply systematic change in risk. However, if poachers destroy evidence before or soon after killing a marked animal, then the situation changes entirely. We refer to these occasions as “cryptic poaching.” Destruction of evidence is rarely, if ever, associated with nonhuman causes of death. We examine the many factors that may lead to an unknown fate in Supplementary Data SD1, but in the section below, we focus on cryptic poaching. We treat cryptic poaching as an event with estimable frequency. Attempting to estimate the causes of death of the unknown fates can be important if poachers commonly destroy evidence or poaching is common. Therefore, we present approaches to confront that challenge in estimation.

First, we consider and reject 2 extreme approaches to estimating the expected values in Table 1B and *P* for cryptic poaching. By rejecting the extreme approaches, we clarify the more credible intervals around the values of interest. One extreme approach inspired by cryptic poaching might be to apportion all the unknown fates to other human causes in Table 1B and none to nonhuman causes, assuming that unknown fates only arise from a human destroying evidence. That approach certainly exaggerates poaching, because technology failure, and marked animals that elude monitoring but later die of nonhuman causes, can lead to some disappearances (Supplementary Data SD1). Likewise, the alternative extreme would apportion all unknown fates to nonhuman causes and none to other human causes. That assumption requires more evidence to reject, which we present in Supplementary Data SD1. Nevertheless, the extreme (no cryptic poaching) is illogical by our definition of an animal that eludes monitoring. That some marked animals live and die unmonitored is likely, but eluding monitoring does not immunize animals from poaching unless all poachers avoid marked animals. That seems infeasible if traps, poison, or shooting under conditions of low visibility occur. Therefore, the second extreme approach is also unrealistic. We assume cryptic poaching occurs and we present 2 reasonable approaches to estimate the expected values in Table 1B.

One reasonable approach to estimate cryptic poaching would be to estimate *Expected*_non_ (the number of marked animals of unknown fate expected to die from nonhuman causes) and *Expected*_oh_ + *P* (the number of marked animals of unknown fate expected to die from other human causes) by their relative proportions in the known fates, but importantly, excluding legal kills from that calculation. This “equal apportionment approach” perpetuates the assumption that known fates can be extrapolated to unknown fates without further correction than performed in Table 1A. Equal apportionment is appropriate to situations in which 3 criteria are met: 1) marked animals were selected randomly from the population as a whole, 2) marked animals disappear without regard to the cause of death, and 3) the researchers have evidence that marking and monitoring do not affect risk of different causes of death. We predict these conditions will never be met for controversial wildlife, such as wolves, but we provide the approach for other species and for Bayesian modelers who wish to define informative credible intervals. Figure 2A depicts the equal apportionment approach.

**Fig. 3.**
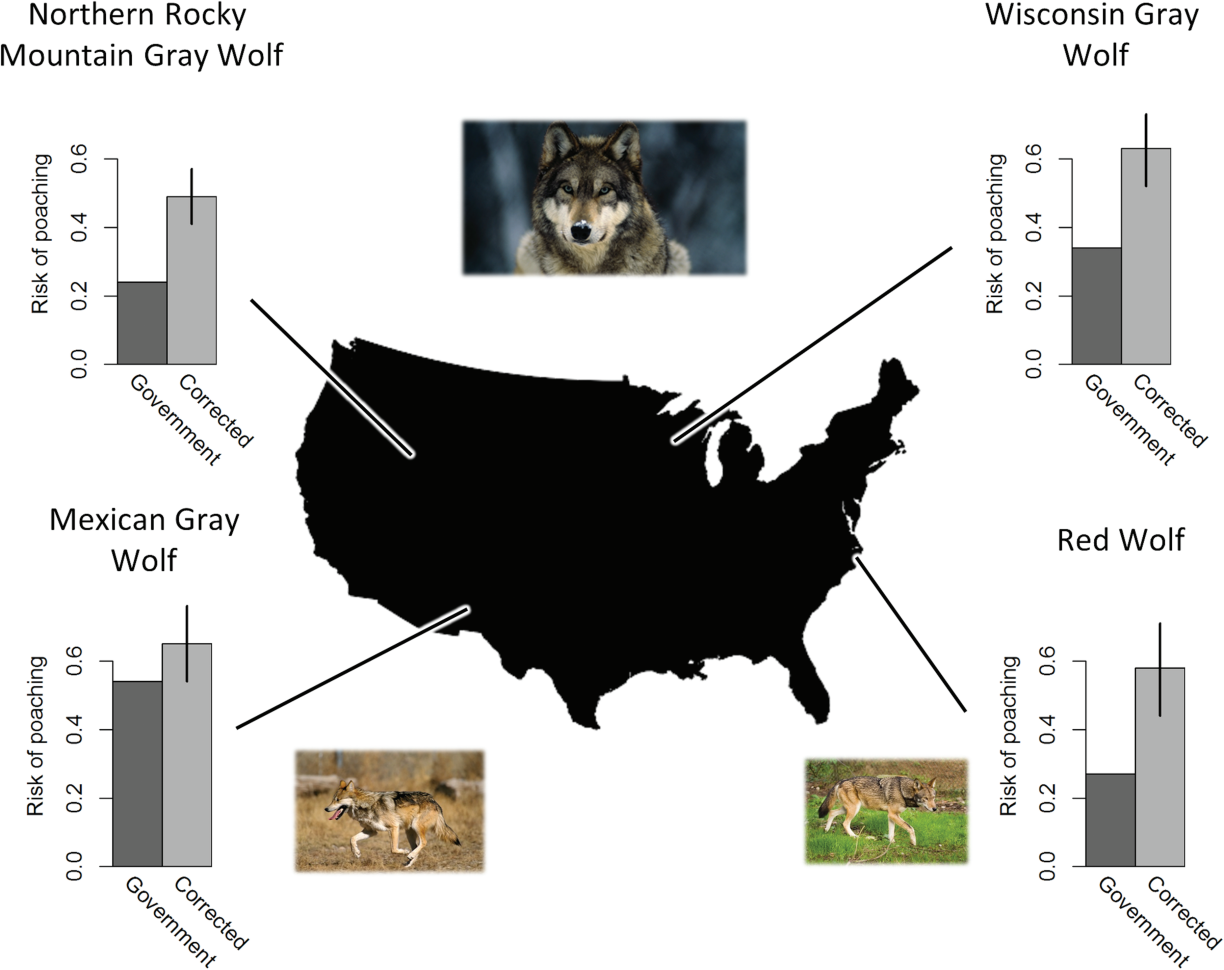
Endangered wolves (gray: *Canis lupus*, Mexican gray: *C. l. baileyi*, and red: *C. rufus*) and risk of mortality from poaching as a proportion of all deaths. Approximate geographic locations are shown for 4 populations in the United States. The relative risks of mortality from poaching by government estimates (dark gray bars, no uncertainty estimates available) are paired with the same estimates from this study (light gray bars; error bars: lower bound derived from the equal apportionment approach and upper bound derived from the Scandinavian estimate of cryptic poaching *C* = 2). See Supplementary Data SD2 for poaching values separated from other human causes: Wisconsin (Natural Resources Board 2012); Northern Rocky Mountain (NRM): (Murray et al. 2010; Smith et al. 2010); Mexican: (USFWS 2015: table 4); red (USFWS 2007: figure 7).

If cryptic poaching is non-zero, then poached animals should be deducted from *m* before equal apportionment occurs, because poachers interrupted monitoring. Cryptic poaching alters estimates of mortality risk because data are lost; more so as concealment behavior spreads or becomes more effective. We have 2 published estimates of cryptic poaching rates to draw upon. For Scandinavian wolves, the cryptic poaching rate was estimated at 66% of total poaching, suggesting that for each observed poached wolf, 2 poached wolves eluded observation (Liberg et al. 2012). For Wisconsin wolves, the corresponding estimate was 46–54% of total poaching (Treves et al. 2017b), or for each observed poached wolf, 1 poached wolf eluded observation (Fig. 2B). In Supplementary Data SD1, we explain why the Wisconsin estimate is conservative. In brief, it treats poaching that was known as if there was no attempt at cryptic poaching. Estimates of cryptic poaching are probably landscape-specific and perhaps specific to certain years because they may reflect accessibility to habitat, human attitudes toward current policy, reporting animal deaths, etc. To isolate poaching from other human causes of death for Figs. 2B and 3, we accepted the official estimates of known-fate poaching and vehicle collisions and applied their ratio to our estimates of other human causes in Table 1B (see Supplementary Data SD2 for the raw data). Then, we used 2 equations to estimate the numbers of marked animals of unknown fates expected to die from nonhuman causes and other human causes respectively, as follows:

$$Expected_{non}=\;(m\;-\;P)•Observed_{non}/(Observed_{non}+Observed_{oh})$$ (1a)

$$Expected_{oh}=\;(m\;-\;P)•Observed_{oh}/(Observed_{non}+Observed_{oh})$$ (1b)

where *n* and *m* are defined above and in Table 1A, *Legal* is the number of marked animals killed legally, *Observed*_non_ is the number of marked animals of known fate that died from nonhuman causes, *Observed*_oh_ is the number of marked animals of known fate that died from human causes other than legal killing, and *P* is defined by equation 2:

$$P\;=\;Poached_{o}•C$$

where *Poached*_o_ is the number of marked animals of known fate that died from poaching and *C* is the scalar of cryptic poaching, which we assigned the values of 0 (equal apportionment), 1 (Wisconsin estimate), or 2 (Scandinavian estimate) as explained above.

## Results

### 

#### Section 1: overestimating risk for perfectly documented causes of death.

Estimating relative risk of mortality from legal causes using only the known fates produced estimates that were 0.05–0.16 higher than when unknown fates were included (Table 2). Published estimates of the risk of legal killing also tend to be higher than ours in Table 2. For Wisconsin wolves, Stenglein et al. (2015) reported 0.125 risk for “*Legal*” (their Table 2), which was 0.063 higher than our estimate for the same period. For NRM wolves, Smith et al. (2010) reported 0.30 risk of mortality from “legal causes,” which is 0.06 higher than our estimate of the risk of mortality from legal causes for the same period. Disparities were not so clear for Mexican and red wolves. Because the USFWS reported mortality risk for Mexican wolves after excluding most legal causes (USFWS 2016c), their proportions are not directly comparable to ours. For red wolves, the USFWS and (Murray et al. 2015) estimated risk as we did (USFWS 2007) citing Murray unpublished. However, disparities between the 2 reports for red wolves could not be reconciled so we used the median which was 0.05 higher than our estimate in Table 2. The overestimates of legal killing in Table 2 increased from 0.05 to 0.16 as the risk of legal killing rose (Fig. 1B).

**Table 2.** Relative risk of mortality from legal killing, as a proportion of all radiocollared wolves (*Canis lupus* or *C. rufus*) that had known fates or unknown fates (disappeared or unknown cause of death) for 4 wolf populations with *n* (number of known fates), *m* (number of unknown fates), and *Legal* (number of marked animals killed legally). NRM = Northern Rocky Mountains.

Population^a^	Known fates (*n*)	Unknown fates (*m*)^b^	Known + unknown fates (*n* + *m*)^b^
Wisconsin gray	0.12	0	0.06
NRM gray	0.40	0	0.24
Mexican gray	0.33	0	0.25
Red	0.13	0	0.08

^a^Wisconsin 1979–2012 *n* = 221, *m* = 210, *Legal* = 27 (Treves et al. 2017b) from their Table 2; NRM 1982–2004 *n* = 320, *m* = 206, *Legal* = 128 (Murray et al. 2010) from their Table 2; Mexican 1998–2015 *n* = 155, *m* = 53 (8 unknown, 6 awaiting necropsy, 39 lost signals), *Legal* = 51 (including permanent removals, and “Other causes of death include capture-related mortalities and legal shootings by the public”), from USFWS (2015); Siminski (2016); USFWS (2016a, 2016c, 2016b, 2016d); North Carolina red wolves 1999–2007 *n* = 111, *m* = 55, *Legal* = 22 “management” (USFWS 2007) citing Murray, unpublished; however, Murray et al. (2015) reported *n* = 91, *m* = 58, *Legal* = 5. We report the median of the 2 red wolf values.

^b^Because legal kills must be reported (known fates) or they are not legal, the corrected risk of legal killing followed the method in Table 1A and Fig. 1A.

#### Section 2: underestimating risk for inaccurately documented causes of death.

Complementary to overestimates of legal killing, estimates of the relative risk of other human-caused mortality using known fates produced lower estimates than when unknown fates were included (Figs. 2A and 2B; Table 3). Official estimates of other human causes of mortality for Wisconsin wolves (Natural Resources Board 2012; Stenglein et al. 2015) were 0.17–0.36 lower than ours in Table 3. The official estimates of risk of mortality from other human causes for NRM wolves from Murray et al. (2010) and Smith et al. (2010) were 0.14–0.27 lower than ours in Table 3. The official estimate of risk of mortality from other human causes for Mexican wolves was 0.07–0.21 lower than ours in Table 3, when calculated with all deaths and permanent removals (USFWS 2016c). The median of the 2 estimates of risk of mortality from other human causes for red wolves was 0.26–0.40 lower than ours in Table 3. Even with the conservative equal apportionment approach, our ranges of estimates all fall above official point estimates made by agencies and biologists.

**Table 3.** Relative risk of mortality from inaccurately documented causes of death, as a proportion of all radiocollared wolves (*Canis lupus* or *C. rufus*) that had known fates or unknown fates (disappeared or unknown cause of death) for 4 wolf populations: *n* (number of known fates), *m* (number of unknown fates), *Observed*_oh_ (number of marked animals of known fate that died from human causes other than legal killing), *Expected*_oh_ (the number of marked animals of unknown fate expected dead from other human causes), *C* is the cryptic poaching scalar of 0, 1, or 2, and *P* is the number of marked animals of unknown fate expected dead from cryptic poaching following equation 2. NRM = Northern Rocky Mountains.

Populations and estimation approaches (*C*)^a^	*Observed*_oh_/*n*	(*Expected*_oh_*+ P*)/*m*	Weighted average
Wisconsin equal apportionment (0)	0.57	0.65	0.60
Wisconsin cryptic poaching (1, 2)	0.57	0.80, 0.95	0.68, 0.75
NRM equal apportionment (0)	0.37	0.61	0.46
NRM cryptic poaching (1, 2)	0.37	0.77, 0.94	0.53, 0.59
Mexican equal apportionment (0)	0.52	0.77	0.59
Mexican cryptic poaching (1, 2)	0.52	1.05, 1.33^d^	0.66, 0.73
Red equal apportionment (0)	0.65	0.74	0.68
Red cryptic poaching (1, 2)	0.65	0.94, 1.13^d^	0.75, 0.82

^a^Sources are identical to Table 2 and raw data are found in Supplementary Data SD2. We used the median of the 2 red wolf values: *Poached*_o_ = 45 (“Private Trap,” “Poison,” “Gunshot”^b^) or 39 (“Gunshot,” “illegal”^c^), *Observed*_oh_ = 23 for both sources^b,c^, comprising 0.76^b^ or 0.72^c^ of *n* − *Legal* = 90^b^ or 86^c^, as the number of marked animals killed legally.

^b^USFWS (2007).

^c^Murray et al. (2015).

^d^Values exceeding 1.0 arose when equation 2 yielded a higher value than *m*.

Poaching in particular has been underestimated systematically by biologists and policy makers (Fig. 3). In Fig. 3, we present the official estimates of poaching for 4 endangered wolf populations in the United States, compared to our range of estimates from Table 3 and Supplementary Data SD2. Using the Wisconsin estimate of cryptic poaching (50%), our estimates of risk of mortality from poaching are 0.17–0.32 higher than official estimates of the risk of mortality from poaching. The Scandinavian estimate of cryptic poaching (66%) yielded estimates of risk of mortality from poaching that are 0.32–0.45 higher than official estimates of the risk of mortality from poaching. The Wisconsin estimate of cryptic poaching lies near, but slightly higher, than the median between the equal apportionment lower bound (Fig. 2A) and the Scandinavian cryptic poaching upper bound (Fig. 2B), which suggests slightly asymmetrical credible intervals because of negative skew.

Supplementary Data SD3 presents our estimates of risk of mortality for 3 causes of death (see Supplementary Data SD2). Poaching was the major cause of death for the 4 endangered wolf populations.

## Discussion

The relative risks of different causes of death for marked animals have often been miscalculated under 1 or both of the following common conditions: 1 or more causes of death were perfectly reported but others were not, or marked animals had unknown fates (i.e., disappeared without a trace or were recovered but the cause of death was undetermined). The resulting bias overestimates the perfectly reported causes of death, such as legal killing, and underestimates the others, such as poaching. With evidence from 4 endangered wolf populations in the United States, we showed the miscalculation biased estimates substantially upwards for legal killing and biased them substantially downwards for other human causes (mainly poaching and vehicle collisions; Fig. 3 and Supplementary Data SD3). The error is non-random (systematic bias) and will increase under several common conditions: high rates of legal killing (Fig. 1B), high proportions of unknown fates (Fig. 2A), and high rates of cryptic poaching (i.e., unreported killing associated with destruction of evidence; Fig. 2B).

The corrections we applied, under even the most conservative equal apportionment approach, yielded estimates indicating that unregulated human-caused mortality was the major cause of death in endangered wolf populations in the United States (Supplementary Data SD3). Observed poaching in all the populations we studied outnumbered the primary other human cause of death, vehicle collisions, by a factor of 2 or more. That means most of the underestimation of other human causes was due to underestimating poaching. When we corrected the bias, we found substantial underestimates of poaching (Fig. 3). Indeed, for every wolf population we examined, we found poaching was the greatest threat. In the NRM wolf populations from 1982 to 2004, poaching replaced legal killing as the major threat to wolves after correcting for the mathematical miscalculation of legal killing. For the other wolf populations, the official reports had correctly identified poaching as the major threat, although they underestimated it.

There are several reasons our estimates of poaching are higher than previous ones. First, we demonstrated that prior estimates would have underestimated causes of death that are not perfectly documented. Second, we took 2 approaches to reconstruct the unknown fates of radiocollared wolves. The first approach, equal apportionment, assumes unmonitored wolves die of the same fates at the same rates as monitored wolves. This is unlikely to hold in any population of marked animals, let alone controversial ones such as wolves that are subject to high relative risks of legal and illegal killing. As such, the equal apportionment approach should be seen as a minimum bound on estimates of the risk of mortality from poaching. By contrast, we provided maximum bounds on the estimated risk of mortality from poaching, when we used the cryptic poaching approach, which apportions unknown fates to cryptic poaching first, informed by prior estimates of cryptic poaching from the literature. We used 2 published values for cryptic poaching from the literature (50% and 66%) and found the higher one probably too high (Table 3 footnote d). Accordingly, we recommend the 50% cryptic poaching estimate be used as the median for the likely range of values to estimate the risk of wolf mortality from poaching. These values and approaches may need adjustment for other sites and other species.

The traditional assumption that the causes of death in individuals of known fate are representative of those of unknown fate is inaccurate whenever known fates include both perfectly documented and inaccurately documented causes of death. The bias increases in proportion to the number of legal kills and the number of unknown fates because each one adds additional bias (overrepresenting perfectly documented causes of death and underrepresenting inaccurately documented causes of death, respectively). By accounting fully for all marked animals and by estimating the unknown fates, we can extract more information from the sample of marked animals than has been done traditionally. Extracting more information is desirable from the standpoint of management efficiency (less effort to mark animals is wasted when data are lost) and also for accuracy.

Some authorities will dismiss relative risk estimates as irrelevant for populations perceived to be large, growing, and resilient. Such a dismissal might be biologically inappropriate. Three studies of gray wolves, 1 in Wisconsin and 2 separate populations in Alaska (Schmidt et al. 2015; Borg et al. 2016; Treves et al. 2017b), demonstrate that mortality rates (per capita hazard) for marked wolves were as different as 15–28% from the per capita hazard rate for unmarked wolves. A mechanistic link between mismeasured risk and unrepresentative hazard rates for marked animals might exist. For example, it might relate to the methods used in recent years to mark wolves, such as livetrapping in areas where few people spend time or livetrapping in core areas of established wolf pack territories, both of which may capture individuals with lower exposure to human-caused mortality (Treves et al. 2017b). Alternatively, hunters and poachers may be able to target (or avoid) marked wolves with high accuracy, a possibility that has not been studied from the perspective of hunters and trappers, to our knowledge. If marked and unmarked animals experience differential per capita hazard rates, then marked animals will become less representative of the population as the relative risk of human-caused mortality increases. Such a relationship could account for the empirical observations of accelerating declines in wolf population growth as human-caused mortality increases (Adams et al. 2008; Creel and Rotella 2010; Vucetich 2012).

Pending further study, we advise against extrapolation from data on haphazardly marked animals of any species. Moreover, one should not discard the lost data from marked animals of unknown fate as is common in wildlife mortality analyses (Liberg et al. 2012). We recommend governments and researchers report data on marked and unmarked animals transparently, including “time on the air” for telemetry data. Additionally, spatial variation in human density and activity across the range of marked animals might be useful when poaching is a major cause of death for study subjects. Together, such steps would improve estimates of mortality parameters for marked animals and, consequently, help to avert policy errors.

## Supplementary Data

Supplementary data are available at *Journal of Mammalogy* online.

**Supplementary Data SD1.**—Disappearances of marked animals.

**Supplementary Data SD2.**—Data for calculations in Tables 2 and 3, and Supplementary Data SD3.

**Supplementary Data SD3.**—Revised estimates of risk for each category of cause of death in endangered wolf populations in the United States.

## Supplementary Material

Supplementary_Data
