# Supplementary material for: Mismeasured mortality: correcting estimates of wolf poaching in the United States
Source: J Mammal. 2017 May 19;98(5):1256–64. doi: 10.1093/jmammal/gyx052 (PMC6093422; doi:10.1093/jmammal/gyx052)
Supplement: Supplementary_Data [file gyx052_suppl_supplementary_data.zip › gyx052_suppl_Supplementary_Data_S3_legend.docx]

**Supplementary Data S3**.–– Revised estimates of risk for each category of cause of death in endangered wolf populations in the United States. Approximate geographic locations are shown for four wolf populations. The relative risks of mortality from legal killing, nonhuman causes, vehicle collisions, and poaching include error bars as follows: lower bound derived from the equal apportionment approach and upper bound from the Scandinavian estimate of cryptic poaching *C* =2 (source data in Supplementary Data S2).
