# Supplementary material for: Mismeasured mortality: correcting estimates of wolf poaching in the United States
Source: J Mammal. 2017 May 19;98(5):1256–64. doi: 10.1093/jmammal/gyx052 (PMC6093422; doi:10.1093/jmammal/gyx052)
Supplement: Supplementary_Data [file gyx052_suppl_supplementary_data.zip › gyx052_suppl_Supplementary_Data_S1.docx]

**Supplementary Data S1**.––Disappearances of marked animals

Animals elude monitoring for a variety of reasons. One of the best-understood reasons is the failure of transmitters, batteries, or collars. VHF technology is generally the most reliable telemetry method (Mech and Barber-Meyer 2002), but the technology can still fail. A recent comparison of five manufacturers quantified reliability (Habib et al. 2014): ATS (100% reliable), Wildlife Materials (96%), Telonics (86%), AVM Instruments (58%), and HABIT (39%). The maker of radio-collars was not specified in the government reports we analyzed nor could we find a reference to which brands were sold or deployed most commonly to United States governments. In our experience, the most common brands were ATS, Wildlife Materials, and Telonics. Therefore, we assume an overall 6% failure rate from the average of the three brands as measured by (Habib et al. 2014). We use that value qualitatively below.

Once a marked animal eludes monitoring, the animal may die from any cause, some of which might entail concealment of evidence. Even poaching with no intent to conceal evidence can result in lost data from marked animals that eluded monitoring. For example, in Wisconsin, it was legal to kill a coyote in many locations most of the year without reporting the kill, so it is conceivable that a wolf that had eluded monitoring was killed under mistaken identity by a poacher who did not retrieve the carcass and therefore did not detect the collar. Therefore, technology failure creates detection bias for death from many causes. In addition, some marked animals may elude monitoring if transmitters fail after vehicle collisions, chewing by other animals, or natural causes that bury or damage a transmitter. We cannot at present estimate the frequencies of such events, but again, detection bias is added to a death from any of the typical causes that are not perfectly documented. Therefore, the values in Tables 1B and 3 for *Expected_non_* and *Expected_oh_* contain instances of technology failure. Our assumption above about a 6% technology failure rate and our assumption that collars fail under other circumstances but very rarely suggests that unknown fates should be rare without additional reasons for disappearances of marked animals. Put another way, Supplementary Data S2 and Table 2 reveal that one-third to one-half of marked, radio-collared wolves disappeared. The disparity between that range of values and 6% suggests another reason exists for the disappearances of marked wolves.

That leaves us to consider cases in which an animal eluded monitoring but its technology did not fail. The effort invested in monitoring in both time and area covered would presumably affect the probability that an animal eludes monitoring for long enough to be classified as unknown fate. For example, Wisconsin wolf monitors seemed to stop searching for a missing radio-signal after a few months at most of medium-altitude aerial telemetry (Treves et al. 2017), whereas NRM wolf monitors appeared to use high-altitude aerial telemetry over a wider area (Smith et al. 2010). We are not aware of quantifications of the effort expended or rate of return per unit effort in those two studies. Neither Murray et al. (2010) nor Smith et al. (2010) quantified how many marked NRM wolves eluded monitoring but were found later by other means. Presumably, such values are site-specific and perhaps time-specific By contrast, in Wisconsin, 26% of the marked wolves that had eluded monitoring were later found dead; and found by other means than telemetry. Conversely, 74% of marked wolves eluded monitoring and were never recovered. Further examination of the Wisconsin data suggests the reporting rates for marked wolves that had eluded monitoring but were found dead by other means varied by cause of death. Treves et al. (2017) estimated the reporting rate at 17% for nonhuman causes and 50% for vehicle collisions (Treves et al. 2017). Presumably, deaths on roads were associated with a 33% higher reporting rate because the driver or subsequent passers-by reported the collared carcass to monitors. Therefore, Treves et al. (2017) predicted that the corresponding reporting rate for poached, marked wolves would be lower than that associated with vehicle collisions. Indeed, their reconstruction of unknown fates led to an estimate of reporting rate for poached, marked wolves of 18% (Treves et al. 2017). The similarity of this reporting rate to that for nonhuman causes suggests that recovering marked wolves that eluded monitoring in Wisconsin was as difficult for poached wolves as for those that died of nonhuman causes. We predict therefore that cryptic poaching occurs in areas with low human use (unlike roads).

Inferences about unknown fates of marked animals hinge critically on careful consideration of detection bias and reporting bias. Our method in Table 1B contains an implicit hypothesis about the accuracy of documentation for different causes of death. The three categories of cause of death vary from perfectly documented to two different forms of incomplete documentation (inaccuracy). The first category is legal killing, (reporting bias = 0 and detection bias = 0). The second category includes causes of death that were unrelated to human action, but the monitors lose information because the death is not detected by the same method as known fates (e.g., telemetry). The result is detection bias. The third category includes causes of death that involve humans (i.e., the deaths were detected), yet the monitors lose information because the deaths were not reported (i.e., reporting bias plus detection bias). Reporting bias can arise from concealment of evidence (cryptic poaching or unintentional lack of reporting). For example, a person may be unaware they have killed a marked animal, including vehicle collisions at high speed or in poor light, weapons that lead to death long after an encounter, or mistaken identity between species. All these could stymie reporting of a dead marked animal, even by a person who intends to report or has a permit to kill that animal (Newsome et al. 2015; Treves et al. 2017). Regardless, the animal in those cases ends up as an unknown fate due to reporting bias.

***Cryptic poaching is non-zero*.––**Analysis of ‘time on the air’ for radio-collared Wisconsin wolves revealed that the average interval between dates of collaring and disappearance of 534 days (*SD* 767 days) was similar to that for poached wolves (547 days); by contrast, the average intervals for nonhuman causes and vehicle collisions were 679 and 807 days, respectively (Treves et al. 2017). That makes cryptic poaching seem probable for many unknown fates, although circumstantially. Additionally, veterinary pathology information from necropsy and radiography for some Wisconsin wolves revealed that poaching was missed in 6–37% of cases (depending on which subsets of carcasses were considered), even for ostensibly known fates. One cannot extrapolate from these percentages because the samples were not random, but one can infer that measurement of known fates was biased low for poaching (Treves et al. 2017). Prior and concurrent work on Wisconsin wolf mortality did not report these and other biases (Wydeven et al. 2001; Stenglein et al. 2015). If such measurement errors arise beyond Wisconsin, then the nonhuman causes among known fates contain more poached wolves than the converse.

Among the NRM wolves, the median and average time to disappearance were 96% and 104% of the median and average time to known fates respectively (Smith et al. 2010). They did not provide these data by cause of death but technology failure would seem an unlikely explanation for so many disappearances with similar timing.

Liberg et al. (2012) also presented evidence for suspicious disappearances of marked wolves from an almost completely closed population that was monitored intensively with telemetry and genetic fingerprinting.

Finally, abundant anecdotal claims about poaching and concealing evidence pervade the literature on wolves (reviewed in (Browne-Nuñez et al. 2015). In sum, it is highly unlikely that unknown fates are either all poaching or all non-poaching.

**Literature Cited**

Browne-Nuñez, C., A. Treves, D. Macfarland, Z. Voyles, and C. Turng. 2015. Tolerance of wolves in Wisconsin: A mixed-methods examination of policy effects on attitudes and behavioral inclinations. Biological Conservation 189:59–71.

Habib, B., S. Shrotriya, K. Sivakumar, P. R. Sinha, and V. B. Mathur. 2014. Three decades of wildlife radio telemetry in India: a review. Animal Biotelemetry 2:4:DOI: 10.1186/2050-3385-1182-1184.

Mech, L., and S. M. Barber-Meyer. 2002. A critique of wildlife radio-tracking and its use in National Parks). U.S. National Park Service, Fort Collins, CO.

Murray, D. L. et al. 2010. ￼Death from anthropogenic causes is partially compensatory in recovering wolf populations. Biological Conservation 143:2514–2524.

Newsome, T., J. T. Bruskotter, and W. J. Ripple. 2015. When shooting a coyote kills a wolf: Mistaken identity or misguided management? Biodiversity and Conservation 24:3145-3149.

Smith, D. W. et al. 2010. Survival of colonizing wolves in the Northern Rocky Mountains of the United States, 1982 – 2004. Journal of Wildlife Management 74:620 – 634.

Stenglein, J., T. R. Van Deelen, A. P. Wydeven, D. J. Mladenoff, J. Wiedenhoeft, J. A. Langenberg, and N. J. Thomas. 2015. Mortality patterns and detection bias from carcass data: An example from wolf recovery in Wisconsin. Journal of Wildlife Management 7:1173-1184.

Treves, A., J. A. Langenberg, J. V. López-Bao, and M. F. Rabenhorst. 2017. Gray wolf mortality patterns in Wisconsin from 1979 to 2012. Journal of Mammalogy 98:17-32.

Wydeven, A. P., D. J. Mladenoff, T. A. Sickley, B. E. Kohn, R. P. Thiel, and J. L. Hansen. 2001. Road density as a factor in habitat selection by wolves and other carnivores in the Great Lakes Region. Endangered Species Update 18:110-114.
