# Supplementary figures and images for: Mismeasured mortality: correcting estimates of wolf poaching in the United States
Source: J Mammal. 2017 May 19;98(5):1256–64. doi: 10.1093/jmammal/gyx052 (PMC6093422; doi:10.1093/jmammal/gyx052)

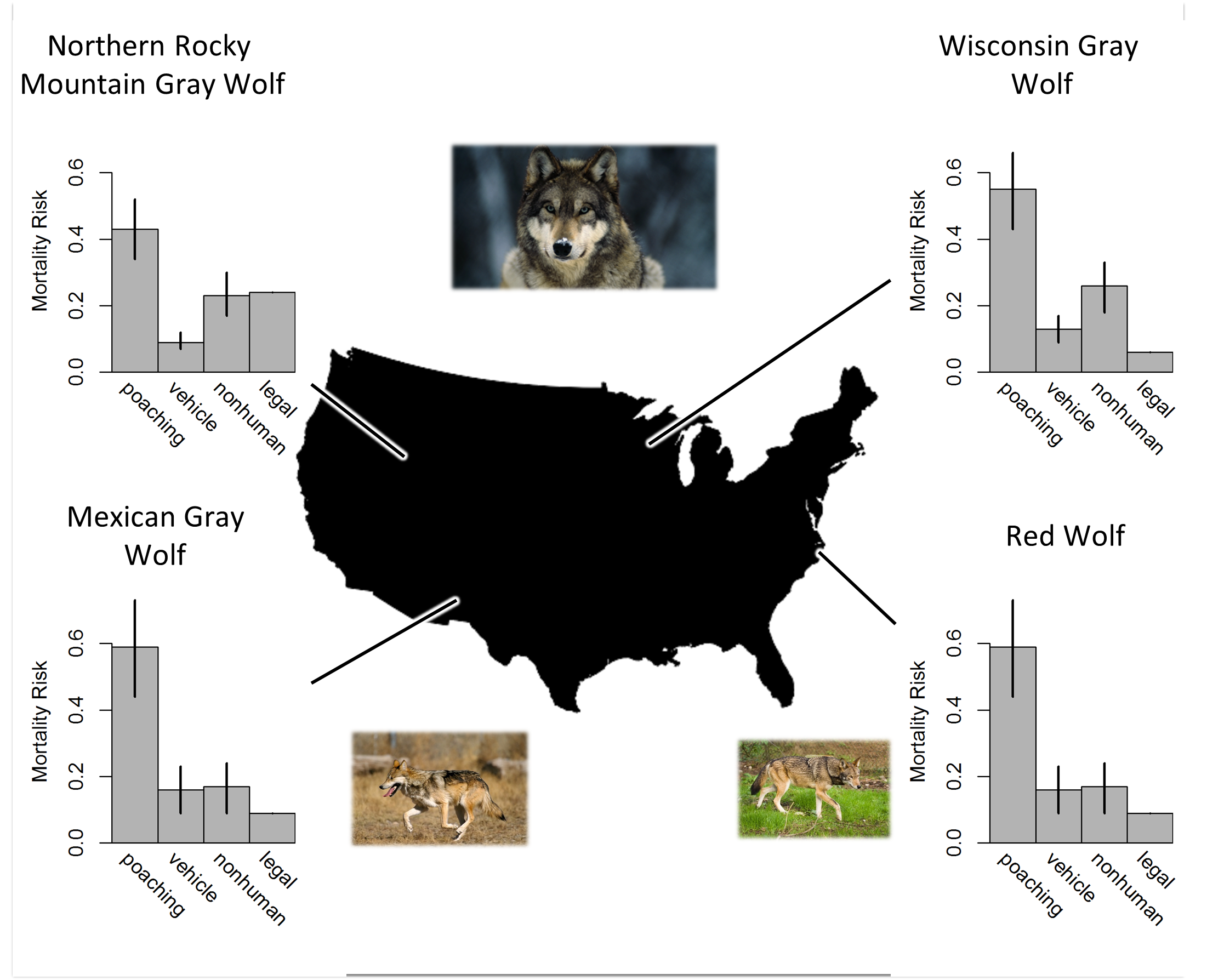

Supplement: Supplementary_Data [file gyx052_suppl_supplementary_data.zip › gyx052_suppl_Supplementary_Data_S3.tif]
